# Into the Future: Fighting Melanoma with Immunity

**DOI:** 10.3390/cancers16234002

**Published:** 2024-11-29

**Authors:** Derek A. Corica, Scott D. Bell, Peyton J. Miller, Daniel T. Kasperbauer, Nicholas J. Lawler, Mark R. Wakefield, Yujiang Fang

**Affiliations:** 1 Department of Microbiology, Immunology & Pathology, Des Moines University, West Des Moines, IA 50266, USA; derek.a.corica@dmu.edu (D.A.C.); scott.d.bell@dmu.edu (S.D.B.); peyton.j.miller@dmu.edu (P.J.M.); daniel.kasperbauer@dmu.edu (D.T.K.); nicholas.j.lawler@dmu.edu (N.J.L.); 2 Department of Surgery, University of Missouri School of Medicine, Columbia, MO 65212, USA; wakefieldmr@health.missouri.edu; 3 Ellis Fischel Cancer Center, University of Missouri School of Medicine, Columbia, MO 65212, USA

**Keywords:** melanoma, melanoma treatment, melanoma immunotherapy

## Abstract

Immunotherapy offers a novel and promising option in the treatment of late-stage melanoma. By utilizing the immune system to assist in tumor destruction, patients have additional options after tumor progression. Immune checkpoint inhibitors reduce the ability for tumors to evade the immune system by inhibiting key surface proteins used to inactivate T-cells. Without these surface proteins, T-cells can induce cytotoxic responses against tumors. Tumor infiltrating lymphocyte therapy is a form of adoptive cell therapy that takes advantage of a small subset of T-cells that recognize and infiltrate tumors. Isolation and rapid expansion of these colonies assist the immune system in mounting a charged response that can induce remission. Tumor vaccines deliver a high dose of unique antigens expressed by tumor cells to the entire body. The introduction of large quantities of tumor antigens upregulates antigen presenting cells and leads to effective activation of the immune system against tumors. Cytokine therapy introduces high amounts of chemical messengers that are endogenous to the immune system and support T-cell expansion. While other methods of immunotherapy exist, immune checkpoint inhibitors, tumor infiltrating lymphocytes, tumor vaccines, and cytokine therapy are commonly used to treat melanoma. Like many other cancer treatments, immunotherapy is not without adverse effects, as toxicities represent a major obstacle. However, immunotherapy has been efficacious in the treatment of melanoma.

## Simple Summary

Melanoma is a particularly aggressive form of skin cancer that is diagnosed worldwide. While most cases of melanoma are diagnosed early and treated properly, patients with delayed diagnoses have a poor prognosis. Likewise, as tumor staging progresses, common therapies lose their effectiveness. This observation punctuates the need for new treatments that are effective in every stage of melanoma, particularly the advanced stages. This review focuses on immunotherapy, a form of treatment that utilizes the immune system to treat melanoma. We aim to summarize and highlight the advances in immunotherapy for the treatment of melanoma with hopes to aid clinicians who are treating melanoma.

## 1. Introduction

Melanoma is a particularly aggressive form of cancer that develops from melanocytes, cells that function to produce melanin, a pigment that provides protection against UV-radiation [1,2]. While highly associated with cancer of the skin, melanoma can develop elsewhere in the body. Cutaneous melanoma, which arises from melanocytes in the epidermis, is the most common site of development, comprising approximately 90% of the overall melanoma diagnoses [2,3]. Other prominent sites of development include the mucosal surfaces of the body and the eye. Mucosal melanoma is typically found in the oral, nasal, and genital mucosa [4], while uveal melanoma develops in the choroid, ciliary body, and iris of the eye [5]. While less common than cutaneous melanoma, both uveal and mucosal melanoma have their own diagnostic challenges. Mucosal melanoma has a very wide range of clinical presentations that is dependent on the mucosal surface involved. This, paired with difficulty in assessing mucosal surfaces, leads to more advanced stages at the time of diagnosis [6,7]. Additionally, approximately half of uveal melanoma cases will have metastasis at the time of diagnosis [8].

The most recent data published by the National Cancer Institute confirm that melanoma diagnoses are on the rise. The prevalence of melanoma in 2021 was 23.8 per 100,000 people, compared to 18.1 per 100,000 in 2000 [9]. Moreover, in Western populations, it is estimated that 1 in every 50 people will be diagnosed with melanoma [10,11]. Fortunately, many cases of melanoma are diagnosed in the early stages and can be surgically removed [12]. Early-stage melanoma (stage I–II) has a 5-year relative survival rate near 100% [9]. However, metastatic melanoma brings a worse prognosis and is more difficult to treat. Moreover, the current 5-year survival for stage III–IV melanoma drops to 35% [9]. This is owed to the ineffectiveness of traditional chemotherapy once melanoma metastasizes [13]. The poor survival rates highlight the need for new and improved treatment modalities that are effective against melanoma once it metastasizes. Two observations that offer promise for therapy are melanoma’s apparent immunogenicity and the immune system’s ability to recognize and infiltrate melanoma cells [14]. This has opened a large body of research into harnessing the immune system to fight melanoma. This review aims to summarize the current advances in immunotherapy for melanoma.

## 2. Cytokine Therapy

Cytokine therapy was the first immunotherapy approved by the United States Food and Drug Administration (FDA) to treat advanced or high-risk melanoma [15]. Cytokine and other immune therapies have provided clinicians with an effective means of treating metastatic melanoma which previously had a very poor prognosis. Before the implementation of immunotherapy, unresectable metastatic disease had a median survival time of 6–9 months [15]. Today, the median survival time of a patient with advanced melanoma is closer to 6 years [16].

Cytokines are small signaling proteins secreted by cells that allow for communication and modulation between cells [17]. They act primarily on the immune system and function to modulate immune function and response [18]. Cytokine therapy treats cancer by amplifying the patient’s immune system to better recognize and destroy cancer cells [18,19].

Interferon alfa-2b was the first immunotherapy approved by the U.S. FDA to treat malignant melanoma. It was approved by the FDA in 1995, but was not widely used as a monotherapy due to its high toxicity and mild improvement in survival [20].

In 2011, the U.S. FDA approved the use of peginterferon alfa-2b (PEG-IFN) for use as adjuvant treatment following definitive surgical resection [21]. A large phase III study of PEG-IFN as an adjuvant therapy conducted by Eggermont et al. determined that prolonged PEG-IFN treatment significantly improved survival when compared to observation alone for patients with stage III melanoma [22]. Patients with distant metastases also saw an improvement in survival with prolonged PEG-IFN treatment, but these results were not statistically significant [22]. Subsequent studies exploring the effectiveness of PEG-IFN produced mixed results regarding its efficacy and improvement in overall survival [23]. Newer studies have demonstrated that PEG-IFN may provide benefit when combined with other immunotherapies such as pembrolizumab or dacarbazine [24,25]. Issues with toxicity and tolerance persist and remain a major constraint in the use of interferon alfa-2b for the treatment of melanoma [21,22].

High-dose interleukin-2 (IL-2) was approved by the U.S. FDA to treat metastatic melanoma in 1998. IL-2 is an immune modulator that stimulates T-cell proliferation and activation [26]. IL-2 treatment is thought to support cytotoxic T-cell expansion, but its precise mechanism has not been well characterized [26]. Cytotoxic T-cells can then target and kill metastatic cancer cells.

There is a variable response among patients receiving high-dose IL-2 treatment for malignant melanoma. Variations in response to IL-2 treatment are likely due to modulation of regulatory T-cells (Tregs) by IL-2 treatment [27,28]. Patients who responded well to IL-2 treatment had minor elevations in Tregs following treatment, whereas patients who did not respond well to IL-2 therapy had significantly elevated Tregs after their first cycle of IL-2 [28]. Therefore, monitoring the level of Tregs during treatment may help predict a patient’s response to high-dose IL-2 treatment. Atkins et al. compiled data from eight clinical trials involving a total of 270 patients to determine the overall effectiveness of high-dose IL-2 treatment [29]. They determined that the overall objective response rate for malignant melanoma was 16%, with 6% of patients achieving complete responses and 10% reaching partial responses following high-dose IL-2 treatment [29].

High-dose IL-2 treatment for malignant melanoma faces similar obstacles to interferon alfa-2b limiting its use. For high-dose IL-2 to work properly, high serum concentrations are required, often resulting in significant toxic effects such as fever, nausea, vomiting, asthenia, and capillary leak syndrome [30]. The significant toxicities and variable Treg responses have limited the effectiveness of high-dose IL-2 therapy and require appropriate critical care support during treatment.

Recently, bempegaldesleukin (BEMPEG), a cytokine prodrug of pegylated interleukin-2, emerged as a new potential treatment for advanced melanoma when combined with the immune checkpoint inhibitor nivolumab [31]. The initial phase II study for this combination was encouraging [31]; however, the phase III PIVOT IO 001 (NCT03635983) trial concluded that BEMPEG plus nivolumab combination therapy did not provide clinical benefit when compared to nivolumab monotherapy [32].

Another potential application of IL-2 therapy that has emerged recently is employing IL-2 as a neoadjuvant to treat malignant melanoma in combination with immune checkpoint inhibitor therapies such as anti-CTLA4 and anti-PD1. A study by Kaptein et al. found that IL-2, anti-CTLA-4, and anti-PD1 triple immunotherapy was able to convert anti-CTLA4 + anti-PD1 non-responders to responders in ex vivo patient melanoma tumors [33]. Other studies have also demonstrated that the addition of IL-2 treatment may improve outcomes in patients not responding well to immune checkpoint therapy [34,35]. Studies have also demonstrated that the overall objective response rate may be improved by combining IL-2 treatment with melanoma vaccines [36].

Antibody-cytokine fusion therapies have also emerged as a promising treatment for malignant melanoma. Recombinant L19 monoclonal antibody bound to IL-2 (L19IL2) allows for targeted delivery of IL-2 to the site of the tumor, reducing systemic toxicities [31,37]. The L19 antibody targets the extra-domain B of fibronectin, which is found on tumor blood vessels, a marker of tumor angiogenesis [38]. This targeting allows cytokine therapy to accumulate and provide long-lasting, local effects [38]. This treatment has been combined with other chemotherapies such as dacarbazine and anti-CTLA-4 to improve tumor responses [39,40]. Another antibody-cytokine fusion, L19TNF, has been combined with L19IL2 to form a drug combination called daromun [38]. A phase III trial conducted by Hauschild et al. (NCT02938299) recently investigated the efficacy of daromun as a neoadjuvant treatment for resectable stage III melanoma [41]. This study found that intralesional neoadjuvant daromun therapy improved relapse-free survival from 6.7 months to 16.7 months when compared to surgery alone [41]. Additionally, neoadjuvant daromun significantly improved distant metastasis-free survival and was not associated with major adverse effects [41].

## 3. Immune Checkpoint Therapy

The viability of immune checkpoint inhibitors (ICI) in their use of melanoma is drastically increasing. With more clinical trials using ICIs, they have potential to broadly become the standard of care in cases of advanced melanoma [42,43]. Already, this therapy is the standard of care in many hospitals for stage IV cutaneous melanoma and is more and more used for stage III patients with high risk. Also, early trials have shown that melanoma variants with and without BRAF-V600E mutation show promising tumor response to ICI therapy [42]. Typically, cancers are capable of masking themselves from the immune system, thus avoiding cell destruction and allowing for enhanced metastases [44]. ICIs are commonly applied in the form of monoclonal antibodies (mAb) that target and inhibit immunoregulatory checkpoint proteins on the surface of T-cells, which act as immune system brakes. Once mAb ICIs bind to immunoregulatory checkpoint proteins, the immune system can then enact cytotoxic killing of cancer cells by T-cells [45]. Among the more studied immune checkpoint axes being targeted for enhanced melanoma cell killing are the cytotoxic T-lymphocyte antigen 4 (CTLA-4) axis and programmed cell death protein 1 (PD-1) with programmed death-ligand 1 (PD-L1) axis [46]. Similarly, ICI regulation of both axes enhances cytotoxic killing by T-cells [47,48]. Studies investigating CTLA-4 and PD-1/PD-L1 are becoming increasingly prevalent, with more clinical trials and research fueling their future use in oncologic treatment [49]. Moreover, it has been shown that progression-free survival (PFS), overall survival (OS), and overall response rate (ORR) are superior in unresectable cutaneous melanoma groups when patients are treated with anti-CTLA-4 and anti-PD-1 ICIs as compared to traditional therapy [50].

**Figure 1:**
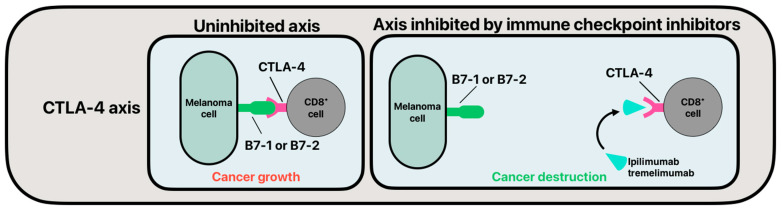
Simplified mechanism of action of monoclonal antibody immune checkpoint inhibitors targeting CTLA-4.

CTLA-4 is a glycoprotein expressed on the surface of T-cells that is related to CD28. CTLA-4 and CD28 can bind to B7-1 or B7-2 on the surface of cancer cells. When CTLA-4 binds to B7-1 or B7-2, the T cell responds by downregulating cell-to-cell cytotoxic effects [51,52]. Conversely when CD28 binds to B7-1 or B7-2 on the surface of cancer cells, T-cell killing is enhanced [53]. Two of the more common ICIs being used to target the CTLA-4 axis are ipilimumab and tremelimumab [50] (Figure 1). Hodi et al. examined the use of ipilimumab with gp100 in patients with unresectable stage III or IV melanoma and found a median OS of 10 months and 6.4 months in the ipilimumab plus gp100 and gp100 only groups, respectively [54]. Further, Robert et al. examined patients with untreated metastatic melanoma by administering ipilimumab and dacarbazine or only dacarbazine and found that OS was longer in the group receiving ipilimumab and dacarbazine versus the group receiving only dacarbazine at 11.2 months and 9.1 months, respectively [55]. Ribas et al. found that overall survival was increased in treatment-naive, unresectable stage IIIc or IV melanoma patients when treated with tremelimumab versus chemotherapy alone [56]. Together, these results substantiate the potential use of CTLA-4 ICIs as the standard of care in cases of advanced melanoma upon future treatment optimization. However, it has been shown that there is a relatively binary response to ICI therapy, since only 20% of patients show disease control after 5–10 years of ipilimumab anti-CTL4 treatment [57]. Thus, finding ways to enhance response in patients treated with ICIs is suspected to be a growing area of scientific interest.

**Figure 2:**
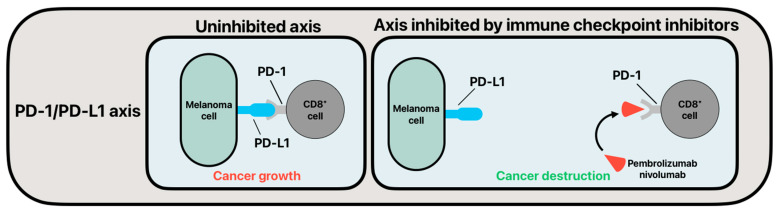
Simplified mechanism of action of monoclonal antibody immune checkpoint inhibitors targeting the PD-1/PD-L1 axis.

The PD-1/PD-L1 axis is an immunoregulatory pathway that results in latent protection of cancer cells when the axis is undisrupted in cancer pathology [58]. PD-1 is a transmembrane protein on the surface of T-cells that attaches to its ligand, PD-L1, on the surface of cells, and more specifically cancer cells in the context of increased cancer survival [59]. When the PD-1/PD-L1 connection is maintained, the immune response is downregulated and self-tolerance mechanisms are enhanced [59]. At the center of melanoma treatment today are novel ICIs that target the PD-1/PD-L1 axis. Specifically, an increased body of clinical trials are being conducted on the PD-1 targeting ICIs, nivolumab and pembrolizumab (Figure 2). Both nivolumab and pembrolizumab are monoclonal antibodies that have shown enhanced efficacy in the treatment of advanced melanoma pathology [50]. Weber et al. found that in patients with unresectable or metastatic melanoma that had progressed after ipilimumab, nivolumab showed an increased objective response rate when compared to traditional chemotherapy [60]. Further, Robert et al. found that in untreated patients who had metastatic melanoma without a BRAF mutation, nivolumab gave superior OS, PFS, and ORR when compared to traditional decarbazine chemotherapy [61]. Ribas et al. found that in patients who were refractory to treatment with ipilimumab, pembrolizumab gave superior PFS and 6-month PFS when compared to traditional chemotherapy (Table 1) [62]. Though the use of PD-1 and PD-L1 antibodies is showing positive response rates while treating melanoma, it should be noted that these therapies are still less effective in some melanoma histological variants, such as uveal melanoma [63]. Also, the response rate to anti-PD-1 ICIs is an area that will likely be receiving increased interest in the future since only 33% of patients treated with the anti-PD-1 pembrolizumab showed a response at the 3-year treatment mark [64].

## 4. Cell-Mediated Therapy

**Figure 3:**
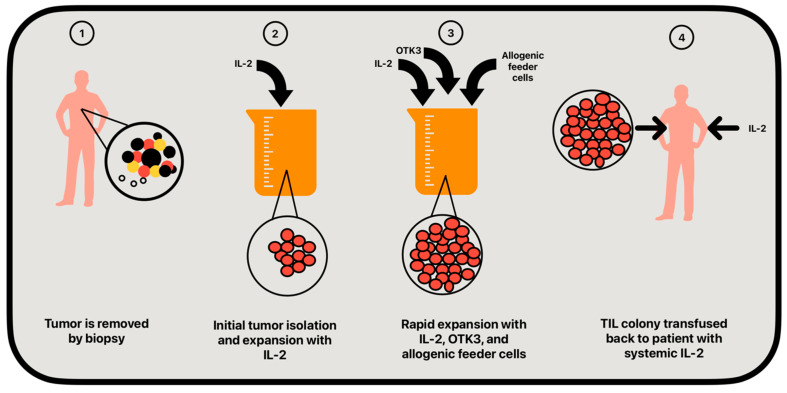
Simplified TIL expansion protocol.

Adoptive cell therapy (ACT) is an emerging area of immunotherapy that offers promise in cancer treatment for both solid tumors and hematological malignancies [65,66]. Tumor-infiltrating lymphocyte (TIL) therapy takes advantage of lymphocytes that can recognize and attack tumor cells. However, limited availability of lymphocytes and the immunosuppressive tumor microenvironment limit the body’s inherent ability to eradicate the tumor [67]. Thus, for treatment to be effective, lymphocyte harvesting and ex vivo expansion is necessary to achieve a cell population that can have therapeutic benefit [68]. The process of TIL preparation consists of an initial growth phase followed by rapid expansion of the cell colony [69]. Initially, the primary tumor or metastases are surgically resected. Tumor material is then shredded into fine particles and incubated with high levels of IL-2, achieving isolation of the initial lymphocyte population [69,70]. Rapid expansion involves incubation with the addition of IL-2, OTK3 (anti-CD3 antibody), and allogenic feeder cells that further activate the T-lymphocyte population. This mixture allows for large-scale expansion of the original lymphocyte population [67,69]. Finally, the solution is administered back to the patient with systemic IL-2 or in combination with other chemotherapeutics (Figure 3) [71,72].

The idea that cell-mediated immunity could play a role in cancer therapy began with studies looking at IL-2, a cytokine that modulates the immune response. Multiple studies demonstrated that IL-2 was able to slow the progression of solid tumors in both animals and humans; this established evidence that the immune system has the capacity to respond to tumor cells [73]. Early studies by Rosenberg et al. demonstrate that TIL, after being removed and amplified in IL-2, slowed the growth of lung and liver metastases in mice [70]. The results of this study prompted further research into the therapeutic potential of TIL. Studies in humans yielded positive results as well: the use of TIL in patients with metastatic melanoma showed a response rate of 35% in patients receiving TIL along with IL-2 and cyclophosphamide [72]. These studies provided a basis for the therapeutic benefit TIL therapy in cancer treatment.

The pioneering studies by Rosenberg et al. showing the benefit of TILs for patients with melanoma have sparked waves of research looking to refine and build upon their initial findings. A study completed by van den Berg et al. demonstrated that patients with stage III melanoma had a 50% response rate to TILs, including two complete responses [74]. Additionally, Besser et al. found that among individuals with stage IV melanoma, positive responders to TILs had a median PFS of 15.43 months compared to non-responders, whose PFS was 2.6 months (*p* < 0.0001) [75]. Moreover, Ngyuen et al. demonstrated a 25% response rate in patients with stage III/IV melanoma who were treated with TIL [76]. These studies, and many others, provided a strong foundation for the use of TILs as an effective treatment for metastatic melanoma.

Larger studies have been conducted to elucidate the role TILs can play in immunotherapy regimens. A study conducted by Sarnaik et al. investigated the potential for TILs to be used as a salvage therapy. Lifileucel, a heterogeneous TIL therapy, was given as a one-time infusion along with a median 5.5 infusions of IL-2 after a lymphodepleting regimen of cyclophosphamide and fludarabine to patients with stage III or IV melanoma who were unresponsive to both ICI therapy and targeted therapy [77]. The results after follow-up showed an overall response rate of 36% (95% CI, 25 to 49) and a disease control rate of 80% (95% CI, 69 to 89) [77]. Resistance to ICI therapy is problematic and treatment options for patients who fail ICI therapy are sparse [78,79]. One of the biggest findings in the Lifileucel study was the observation that TIL can be an effective treatment option in patients who fail ICI therapy.

While the benefit of TIL as a salvage treatment in patients with advanced melanoma who have failed first line immunotherapy has been established, studies have also examined TIL therapy as a monotherapy for the treatment of advanced melanoma. A study conducted by Rohaan et al. compared the efficacy of TIL against ipilimumab, an anti-cytotoxic T-lymphocyte-4 (CTLA-4) antibody in patients with advanced melanoma [80]. At follow-up, patients in the TIL group displayed an average progression-free survival of 7.2 months (95% CI, 4.2 to 13.1) compared to 3.1 months (95% CI, 3.0 to 4.3) in the ipilimumab group [80]. While this study shows TIL therapies’ benefit over those of ipilimumab, further studies are warranted to better understand TIL effectiveness versus the wide spectrum of ICI therapy currently available. The results from such studies would elucidate whether TIL therapy could be used as a first line immunotherapy treatment for melanoma.

Tumor mutation status is an important factor that influences treatment selection for patients with melanoma. BRAF mutations are commonplace in melanoma. Of note, BRAF-V600E mutations are implicated in the growth of melanoma due to activation of downstream MEK/ERK growth pathways [81]. The frequency of this mutation has sparked research investigating the effects of BRAF inhibition on melanoma. Amaria et al. investigated the neoadjuvant BRAF inhibitor dabrafenib and adjuvant MEK inhibitor trametinib against standard of care (SOC) surgical resection plus adjuvant therapy in patients with stage III/IV melanoma harboring BRAF-V600E and BRAF-V600K mutations. The results showed patients in the neoadjuvant group had a PFS of 19.7 months compared to 2.9 months in the SOC group. Additionally, patients in the neoadjuvant group experienced much longer metastasis-free survival compared to the SOC group [82]. Moreover, patients in the neoadjuvant group who achieved a pathologic complete response showed higher levels of CD8+ T-cell infiltration compared to those who did not achieve complete responses [82]. The antitumor response in BRAF-inhibitor-based neoadjuvant therapy is correlated to a strong T-cell response against melanoma tumors. The ability for BRAF inhibition to vigorously stimulate the immune system opens the door for research into combination therapies that combine BRAF inhibition with other immunotherapies. Multiple studies have demonstrated similarities in clinical outcomes of TIL in patients regardless of BRAF mutation status [83,84,85]. These observations suggest TIL can be effective in patients with BRAF mutations who are refractory to other treatments. In fact, the Lifileucel study demonstrated TIL efficacy in patients with BRAF mutations that showed tumor progression while being treated with BRAF inhibitors [77]. Based on these results, combination TIL and BRAF inhibitor therapy could have synergistic effects against melanoma tumors.

Serum biomarkers also play a role in influencing TIL therapy. Levels of lactate dehydrogenase (LDH), a marker for tumor burden, are negatively correlated with clinical response to TIL [86]. Additionally, Seitter et al. demonstrated increasing levels of LDH were associated with a lack of response to TIL [84].

TIL is not without drawbacks. Like many other anticancer therapies, toxicities are a difficult obstacle to navigate and can limit the effectiveness of treatment. Patients receiving TIL therapy commonly suffer from hematological suppression that results from the lymphodepleting regimen before TIL infusion [87,88]. Additionally, patients frequently experience toxicities associated with systemic IL-2 administration after TIL infusion [85,86,87,88]. While reversible, toxicities from both lymphodepletion and IL-2 can require hospitalization for symptom management.

## 5. Tumor Vaccines

### 5.1. History and Introduction

Tumor vaccines are a revolutionary subtype of immunotherapy developed to aid in activating the immune system to target specific tumor cells, as either prophylaxis or therapy to treat ongoing malignancy [89]. The premise behind these vaccines hinges on delivering high quantities of antigens specific to or associated with tumors that are normally upregulated and expressed on cancer cells. Introducing large amounts of these antigens to the body increases the ability of antigen-presenting cells (APCs) as well as CD4+ and/or CD8+ cells to activate, leading to a more effective host immune response [90]. Conveniently, this increased activation of APCs drives increased numbers of CD4+ and CD8+ cells, which can be directly analyzed and used as important biomarkers of tumor vaccine effectiveness. Antigen-presenting cells also produce interferon gamma, another crucial biomarker directly correlated with the population of APCs, which has extreme importance in immune regulation, inflammation, macrophage activation, and antitumor activity. Increased APCs, CD4+, CD8+, and interferon gamma indicate increased tumor vaccine effectiveness. The most promising aspect of tumor vaccines is the variability between vaccine types or platforms that causes this desired response. Currently, the vaccine platforms include peptides or proteins, recombinant vectors, gangliosides, genes, whole tumor cells, and dendritic cells displaying a specific tumor antigen [91,92,93,94,95,96], each offering a potential avenue for a diverse array of vaccines, increasing the number of treatment options for melanoma patients.

Melanoma has been a particular target for tumor vaccines since 1991, with the first being a tumor-associated antigen vaccine targeting MAGE-1. Between 1991 and 2014, many more melanoma tumor-associated antigen and tumor-specific vaccines had been produced but showed no significant clinical effect on melanoma survival [97,98]. Many possible reasons for the decreased effectiveness of tumor vaccines have been postulated, which include but are not limited to the tumor immunosuppressive microenvironment, rapidly mutating surface antigens, downregulation of antigens presented on the cell’s surface, central T-cell tolerance, and ineffective vaccine transport [99,100,101,102,103]. Despite these theories, breakthrough studies have shown significant clinical improvement in patient outcomes.

In 2015, the FDA approved the first melanoma tumor vaccine, called Tamilogene Laherparepvec (T-VEC), which distinctly employs a genetically altered herpes simplex 1 virus. After replacing neurovirulence genes with granulocyte-macrophage colony-stimulating factor (GM-CSF) genes, the resulting virus is injected directly into the tumor, causing lysis and local immune responses secondary to the now GM-CSF-producing cells [104]. The keystone phase III trial compared T-VEC to GM-CSF in patients with unresected stage III and IV melanomas. Among the randomly assigned 436 patients, they found a significant increase in durable response rate with T-VEC (16.3%; 95% CI; 12.1–20.5%) compared to GM-CSF (2.1%; 95% CI: 0–4.5%), overall response rate with T-VEC (26.4%; 95% CI: 21.4–31.5%) versus GM-CSF (5.7%; 95% CI: 1.9–9.5%), and median overall survival for T-VEC being 23.3 months (95% CI: 19.5–29.6 months) and 18.9 months (95% CI: 16.0–23.7 months) for GM-CSF. The most common side effects encountered with T-VEC vaccination were mostly limited to chills, fever, nausea, fatigue, injection-site pain, and flu-like illness. Importantly, no patients expired from the vaccine treatment [105].

### 5.2. Exploring Melanoma Vaccine Platforms

Additional clinical trials on other tumor vaccine platforms are being conducted, like those that focus on tumor-specific antigens, or neoantigens [106]. A phase I clinical trial demonstrating a neoantigen vaccine has used modern sequencing technology to compare exomes between six unique patients’ normal and melanoma cells. Using these differences, 20 neoantigens per patient were identified and utilized in the production of patient-specific vaccines to target each patient’s respective tumor cells. Every patient had previously untreated stage III or IV melanoma and after resection, was given their personalized vaccines over a 20-week duration in a series of primers and boosters. After the vaccination window and the median point of 25 months, the four patients with stage III melanoma had no recurrence of the disease. The remaining two patients with stage IV melanoma and lung metastasis had no radiographic evidence of disease after combination therapy with anti-PD-1 pembrolizumab. At the time of publication for that study, the two patients with metastatic melanoma had ongoing treatment. All treated patients had increased activation of CD4+ and CD8+ T-cells. The evidence in this trial found that their tumor vaccines were efficacious in targeting only tumor cells, eliciting an immune response, and showcasing the feasibility of a personalized melanoma therapy [107,108].

To discuss another avenue of tumor vaccine platform, a phase I clinical trial completed on 89 patients had profound results after conducting a period of vaccinations utilizing a liposomal RNA vaccine targeting melanoma-associated antigens NY-ESO-1, MAGE-A3, tyrosinase, and TPTE. After completion of vaccine treatments, a significant induction of CD4+ and CD8+ T-cells was appreciated as well as effective immunotherapy-induced tumor regression either alone or in combination with a PD-1 inhibitor. Among all the patients in this trial experiencing side effects, the most prominent were fever (82%), chills (71%), headache (37%), fatigue (24%), nausea (22%), and tachycardia (21%) [109]. Unlike other chemotherapeutic agents, this tumor vaccine produces side effects relatively like those of the flu vaccine, which may improve patients’ quality of life during treatment.

More recently, a 2023 double-blind phase IIB clinical trial evaluated the efficacy of an autologous tumor lysate dendritic cell vaccine on extending both disease-free (DFS) and overall (OS) survivals in 187 disease-free stage III and IV melanoma patients. These patients were subdivided into three categories, which determined the vaccine treatment they would receive: placebo, tumor lysate particle-loaded dendritic cells (TLPLDC), and tumor lysate particle-only (TLPO). Patients were evaluated throughout an 18-month vaccination period and their DFS and OS were analyzed at 36 months. Patients who received TLPO had a 36-month DFS of 64% (95% CI: 46–77%) and OS of 94.8% (95% CI: 81–99%). TLPLDC patients had a 36-month DFS of 55.8% (95% CI: 39–69%) and OS of 94.2% (95% CI: 78–99%). Patients who were placed in the placebo group had a DFS of 30% (95% CI: 16–45%) (*p* < 0.001) and OS of 70.9% (95% CI: 52–83%) (*p* = 0.011). Researchers also noted that the patients who received the TLPO and TLPLDC vaccines had only grade one or two adverse events in the treatment arm. These included but were not limited to mild symptoms like injection-site reactions, fatigue, fever, nausea, myalgia, headache, cough, and rash [110].

### 5.3. Melanoma Tumor Vaccines Moving Forward

Clinical trials on unique tumor vaccine platforms like those discussed above are being explored further with hopes that they perform similarly to or better than the FDA-approved T-VEC therapy. Meanwhile, T-VEC therapy has piqued sustained interest since its keystone clinical trial in 2015 with hopes to further improve survival rates. For example, some studies have proposed the cooperative ability of T-VEC with anti-PD-1 in a combined therapy to improve patient outcomes. Specifically, phase I and II clinical trials completed in 2017, 2018, 2021, and 2024 examined T-VEC with anti-PD-1 and had results suggesting the combination has antitumor activity [111]. T-VEC has been postulated to potentially play a role in altering tumor microenvironments, further proposing T-VEC being synergistic or improving the efficacy of other treatment modalities like anti-PD-1 therapy [112,113,114]. This, however, has conflicting evidence with a 2023 phase III randomized double-blind clinical trial of 692 patients with either stage III or IV melanoma. In this study, T-VEC-anti-PD-1 (pembrolizumab) was given to half of the patients while the other half received placebo-pembrolizumab. Comparing the progression-free survival and overall survival between the groups, T-VEC-pembrolizumab produced no significant improvement in either category with *p*-values of 0.13 and 0.74, respectively. The T-VEC-pembrolizumab group did, however, have similar overall safety to T-VEC and pembrolizumab alone [115]. The results of this larger study unfortunately discourage those found in the smaller phased studies using pembrolizumab but do encourage further replication of its results and pursuit of other studies evaluating T-VEC with other anti-PD-1 agents like nivolumab, ipilimumab, and tremelimumab. As previously mentioned, there are quite a few hurdles that impact vaccine efficacy, like the tumor immunosuppressive microenvironment, rapidly mutating tumor surface antigens, and downregulation of tumor antigens presented on the cell’s surface. While not exhaustive, any number of these factors are likely at play, influencing tumor vaccine effectiveness. Moving forward, there are areas of growth that can supplement tumor vaccines, like the example of ongoing studies utilizing gut microbiota to increase efficacy of anti-PD-1 treatment in advanced melanoma patients [116]. While it is just a postulation, this application alongside T-VEC has the potential to produce similar, if not more promising, results than those of T-VEC alone.

BRAF is a biomarker commonly evaluated in the tumor vaccine trials previously discussed, making BRAF mutations a prominent topic in melanoma. Roughly one-third of the patients who received T-VEC-pembrolizumab combination therapy in a large phase III clinical trial had a positive BRAF mutation status. The result of this mutation has been characterized as producing aggressive growth and immunosuppressive environments localized to the tumor, which inhibit tumor vaccine effectiveness. Mutations in the BRAF gene cause about 50% of melanomas, notably BRAF-V600E. These mutations produce antigens that are present on tumor cell surfaces, and thus are targets for tumor vaccines. In 2018, a murine study published results following the use of a BRAF-V600E peptide vaccine on mice positive for the BRAF-V600E mutation. The authors found their vaccine had significant induction of cytotoxic t-lymphocytes, inhibited tumor growth, increased antigen presentation, increased invasion of T-cells into tumor cells, increased macrophage activation, decreased the quantity of immunosuppressive cells, and increased interferon gamma production. Additionally, the mice had no significant weight loss, no organ damage, and normal blood chemistry [117,118]. Recently, a phase I–II clinical trial was conducted on 22 human participants with stage II through IV melanomas. Their vaccine consisted of six non-mutated melanoma-specific helper peptides (6MHP) and an additional BRAF-V600E peptide combined with two agents that enhance APC function and activation, toll-like receptor 3 (TLR3) agonist and CD40 agonist. The study evaluated vaccine efficacy via toxicity and immunogenicity by interferon gamma quantities. The patients experienced only mild grade one and two vaccine-specific side effects and their results supported sufficient immunogenicity [119]. These results suggest the potential for these therapies to be used alongside tumor vaccines in order to mitigate factors that lead to decreased vaccine effectiveness.

## 6. Conclusions

The evolution of immunotherapy has rapidly transformed the treatment landscape for melanoma, a cancer that historically has had a poor prognosis. A wide variety of immunotherapies are available that provide opportunities to leverage the body’s immune system and design new treatment approaches for late-stage melanoma. Immune checkpoint inhibitors, adoptive cell transfer, tumor vaccines, and cytokine therapy are among the most used immunotherapies. Immune checkpoint inhibitors such as anti-CTLA-4 and anti-PD-1/PD-L1 therapies are designed to enhance T-cell killing of tumor cells. These therapies have shown clear efficacy in promoting durable responses and prolonging survival in patients with advanced disease. However, challenges remain, such as treatment resistance and adverse effects like immune-related toxicities.

TILs provide a very personalized therapy option. This treatment is designed to enhance immune cells’ ability within a patient to identify and destroy cancer cells. Success with TIL has been promising, revealing a slowing of progression in many patients with high-grade melanoma. While not all patients were responsive, patients who were responsive showed a significant increase in median progression-free survival overall, and an increase when compared to immune checkpoint inhibitors. TIL is not without side effects: patients who received this treatment most frequently experienced symptoms of hematological suppression that results from the lymphodepleting regimen before TIL infusion. In addition, patients also experienced toxicities associated with systemic Il-2 administration.

Tumor vaccines are another immunotherapy treatment method that have demonstrated promise in generating a more efficacious immune response by delivering colony-stimulating factors or presentation of tumor-associated antigens. New advances in this modality including T-VEC, an oncolytic virus, have demonstrated increased durable response rates by integration and delivery of colony-stimulating factor GM-CSF. Additionally, the use of liposomal RNA vaccines targeting melanoma-associated antigens NY-ESO-1, MAGE-A3, tyrosinase, and TPTE, have shown efficacy in melanoma tumor regression as monotherapy or in combination. Liposomal RNA vaccines rely on enhanced recognition and activation of the immune system from presentations of these antigens. Tumor vaccines are emerging as a promising strategy to personalize treatment for patients. The use of unique neoantigens provides specificity that has the potential to enhance treatment and reduce collateral effects. Tumor vaccines have shown side effects such as fever, chills, and headache but this side effect profile may be preferred by patients compared to other therapies.

Cytokine therapies such as interferon-a2b and interlukin-2 were some of the first FDA-approved immunotherapy treatments for melanoma. Cytokines can modulate immune system activation that has shown variable responses in melanoma patients. Their use may be narrower due to toxicities including fever, nausea, vomiting, asthenia, and capillary leak syndrome. However, there remains a valuable use for cytokine therapies in combination with newer immunotherapeutic agents. Combination with these therapies such as checkpoint inhibitors or vaccines may help limit resistance mechanisms and enhance the efficacy of treatment.

One important consideration for immunotherapy treatment is the risk of relapse in melanoma patients. Roccuzzo et al. observed that while immunotherapy can provide benefits over an extended period of time, patients being treated with this modality are prone to higher relapse rates during their treatment course. This is in contrast to targeted therapy combinations, where higher relapse rates were observed later in a patient’s treatment journey, specifically after treatment concluded [120]. Additionally, targeted combination therapy has been found to be particularly successful in preventing relapse and death in certain melanoma patients, achieving 20% lower risk of death with stage III patients and 25% lower BRAF V600E mutation patients compared to the placebo [121]. While the exact mechanism behind relapses over different time frames is unknown, it may be linked to immunotherapy treatment’s delay in immune system activation. In contrast, treatments using targeted therapies are able to establish faster and more localized control.

Looking forward, combinations of these different immunotherapies with targeted therapies, radiation, and other modalities could improve melanoma patient outcomes by limiting immune system evasion mechanisms and enhancing tumor cell recognition. An important investigation for ongoing research will include the identification of biomarkers that are able to predict the response of treatment. Furthermore, investigations into immune cell population types and tumor environment characteristics could aid in predicting this response. This focus, along with identifying therapeutic treatments that can avoid known toxicities of current regiments, could lead to more personalized and efficacious treatment plans. Continued innovation in immunotherapy provides the opportunity to transform the future of melanoma therapy and significantly improve patient outcomes.

## Figures and Tables

**Table 1:** Various clinical trials investigating immune checkpoint inhibition of the CTLA-4 and PD-1/PD-L1 axes in cutaneous melanoma. Adapted from Yun et al. [50].

Clinical Trial Identification/Reference	Antibody/Target	Clinical Setting	Treatment Setting	Outcome(s)
NCT00094653/Hodi et al. [54]	Ipilimumab/CTLA-4	Patients with unresectable stage III or IV melanoma, whose disease had progressed while they were receiving therapy for metastatic disease.	Ipilimumab was administered with or without gp100 every 3 weeks for up to four treatments. Eligible patients could receive reinduction therapy.	The median overall survival was 10.0 months among patients receiving ipilimumab plus gp100, as compared with 6.4 months among patients receiving gp100 alone. The median overall survival with ipilimumab alone was 10.1 months.
NCT00324155/Robert et al. [55]	Ipilimumab/CTLA-4	Patients with previously untreated metastatic melanoma	Ipilimumab was administered plus dacarbazine or dacarbazine plus placebo, given at weeks 1, 4, 7, and 10, followed by dacarbazine alone every 3 weeks through week 22.	Overall survival was significantly longer in the group receiving ipilimumab plus dacarbazine than in the group receiving dacarbazine plus placebo at 11.2 months vs. 9.1 months, respectively. There were higher survival rates in the ipilimumab–dacarbazine group.
NCT00257205/Ribas et al. [56]	Tremelimumab/CTLA-4	Patients with treatment-naive, unresectable stage IIIc or IV melanoma.	Patients were given tremelimumab or physician’s choice of standard-of-care chemotherapy as either temozolomide or dacarbazine.	The median overall survival by intent to treat was 12.6 months for tremelimumab and 10.7 months for chemotherapy. Objective response rates were 10.7% in the tremelimumab arm and 9.8% in the chemotherapy arm.
NCT01721746/Weber et al. [60]	Nivolumab/PD-1	Patients had unresectable or metastatic melanoma and progressed after ipilimumab or ipilimumab and a BRAF inhibitor if they were BRAF(V 600) mutation-positive.	Nivolumab or investigator’s choice, being dacarbazine or paclitaxel combined with carboplatin, was administered.	Confirmed objective responses were reported in 38/120 patients in the nivolumab group versus 5/47 patients in the investigator’s choice group.
NCT01721772/Robert et al. [61]	Nivolumab/PD-1	Previously untreated patients who had metastatic melanoma without a *BRAF* mutation to receive nivolumab.	Received nivolumab and dacarbazine-matched placebo every 3 weeks or dacarbazine and nivolumab-matched placebo every 2 weeks.	At 1 year, the overall rate of survival was 72.9% in the nivolumab group, as compared with 42.1% in the dacarbazine group. The median progression-free survival was 5.1 months in the nivolumab group versus 2.2 months in the dacarbazine group. The objective response rate was 40.0% in the nivolumab group versus 13.9% in the dacarbazine group.
NCT01704287/Ribas et al. [62]	Pembrolizumab/PD-1	Patients had confirmed progressive disease within 24 weeks after two or more ipilimumab doses and, if BRAF(V600) mutant-positive, previous treatment with a BRAF or MEK inhibitor or both.	Patients received intravenous pembrolizumab or investigator-choice chemotherapy as paclitaxel plus carboplatin, paclitaxel, carboplatin, dacarbazine, or oral temozolomide.	Progression-free survival was improved in those administered pembrolizumab compared to chemotherapy. Six-month progression-free survival was 34% in the pembrolizumab 2 mg/kg group, 38% in the 10 mg/kg group, and 16% in the chemotherapy group.
